# Supplementary material for: SCancerRNA: Expression at the Single-cell Level and Interaction Resource of Non-coding RNA Biomarkers for Cancers
Source: Genomics Proteomics Bioinformatics. 2024 Mar 11;22(3):qzae023. doi: 10.1093/gpbjnl/qzae023 (PMC12016560; doi:10.1093/gpbjnl/qzae023)
Supplement: qzae023_Supplementary_Data [file qzae023_supplementary_data.zip › Supplementary material captions.docx]

**Supplementary material**

**Table S1** **Tissue distribution of ncRNA biomarkers collected in SCancerRNA**

**Table S2 Experimentally supported biological functions and clinical applications of the five types of ncRNA biomarker**
