# Supplementary material for: SCancerRNA: Expression at the Single-cell Level and Interaction Resource of Non-coding RNA Biomarkers for Cancers
Source: Genomics Proteomics Bioinformatics. 2024 Mar 11;22(3):qzae023. doi: 10.1093/gpbjnl/qzae023 (PMC12016560; doi:10.1093/gpbjnl/qzae023)
Supplement: qzae023_Supplementary_Data [file qzae023_supplementary_data.zip › Table S1-done.docx]

| **Table S1 Tissue distribution of ncRNA biomarkers collected in SCancerRNA** | | | | | |
| --- | --- | --- | --- | --- | --- |
| **Tissue** | **lncRNA** | **miRNA** | **circRNA** | **snoRNA** | **piRNA** |
| Bladder | 274 | 5 | 10 | 0 | 1 |
| Blood | 296 | 8 | 4 | 0 | 1 |
| Bone | 333 | 12 | 11 | 0 | 0 |
| Brain | 625 | 1 | 4 | 1 | 0 |
| Breast | 761 | 26 | 14 | 14 | 9 |
| Cervix | 356 | 4 | 10 | 1 | 0 |
| Colorectal | 873 | 54 | 14 | 6 | 11 |
| Endometrium | 87 | 0 | 1 | 0 | 0 |
| Head & neck | 520 | 3 | 19 | 0 | 0 |
| Kidney | 233 | 3 | 10 | 8 | 8 |
| Liver | 1101 | 15 | 29 | 7 | 0 |
| Lung | 1086 | 19 | 27 | 13 | 2 |
| Lymph | 44 | 2 | 1 | 0 | 1 |
| Ovary | 349 | 11 | 12 | 0 | 0 |
| Pancreas | 287 | 2 | 2 | 0 | 0 |
| Prostate | 349 | 21 | 2 | 2 | 0 |
| Skin | 108 | 0 | 1 | 0 | 0 |
| Stomach | 856 | 36 | 37 | 2 | 11 |
| Testis | 10 | 0 | 0 | 0 | 0 |
| Thyroid | 257 | 5 | 8 | 0 | 2 |
| Uterus | 2 | 0 | 0 | 0 | 0 |
| Other | 499 | 23 | 7 | 0 | 1 |

*Note*: ncRNAs, non-coding RNAs; lncRNA, long non-coding RNA; miRNA, microRNA; circRNA, circular RNA; snoRNA, small nucleolar RNA; piRNA, PIWI-interacting RNA.
