# Supplementary material for: SCancerRNA: Expression at the Single-cell Level and Interaction Resource of Non-coding RNA Biomarkers for Cancers
Source: Genomics Proteomics Bioinformatics. 2024 Mar 11;22(3):qzae023. doi: 10.1093/gpbjnl/qzae023 (PMC12016560; doi:10.1093/gpbjnl/qzae023)
Supplement: qzae023_Supplementary_Data [file qzae023_supplementary_data.zip › Table S2-done.docx]

| **Table S2** **Experimentally supported biological functions and clinical applications of the five types of ncRNA biomarkers** | | | | | | |
| --- | --- | --- | --- | --- | --- | --- |
| **Function** | | **lncRNA** | **miRNA** | **circRNA** | **snoRNA** | **piRNA** |
| Biological Function | Cell proliferation/cell growth | 2479 | 38 | 136 | 17 | 12 |
|  | Apoptosis/autophagy | 2686 | 9 | 60 | 9 | 7 |
|  | EMT | 1052 | 6 | 17 | 5 | 3 |
| Clinical Application | Circulating | 1152 | 134 | 8 | 0 | 10 |
|  | Survival | 4223 | 42 | 61 | 38 | 19 |
|  | Recurrence | 294 | 7 | 5 | 3 | 4 |
|  | Migration/metastasis | 3150 | 85 | 159 | 18 | 22 |

*Note*: EMT, epithelial–mesenchymal transformation.
